# Effects of Pope Francis’ Religious Authority and Media Coverage on Twitter User’s Attitudes toward COVID-19 Vaccination

**DOI:** 10.3390/vaccines9121487

**Published:** 2021-12-16

**Authors:** Arkadiusz Gaweł, Marzena Mańdziuk, Marek Żmudziński, Małgorzata Gosek, Marlena Krawczyk-Suszek, Mariusz Pisarski, Andrzej Adamski, Weronika Cyganik

**Affiliations:** 1 College of Applied Informatics, University of Information Technology and Management in Rzeszow, 2 Sucharskiego Str., 35-225 Rzeszow, Poland; agawel@wsiz.edu.pl; 2 Medical College, University of Information Technology and Management in Rzeszow, 2 Sucharskiego Str., 35-225 Rzeszow, Poland; mmandziuk@wsiz.edu.pl (M.M.); mkrawczyk@wsiz.edu.pl (M.K.-S.); wcyganik@wsiz.edu.pl (W.C.); 3 Faculty of Theology, University of Warmia and Mazury in Olsztyn, ul. Oczapowskiego 2, 10-719 Olsztyn, Poland; marek.zmudzinski@uwm.edu.pl; 4 Institute for Education Analysis, College of Media and Social Communication, University of Information Technology and Management in Rzeszow, 2 Sucharskiego Str., 35-225 Rzeszow, Poland; mgosek@wsiz.edu.pl; 5 College of Media and Social Communication, University of Information Technology and Management in Rzeszow, ul. Sucharskiego 2, 35-225 Rzeszow, Poland; mpisarski@wsiz.edu.pl

**Keywords:** pope francis, COVID-19, SARS-CoV2, vaccines, Twitter, sentiment analysis, media and communication studies, catholic theology, religion leadership, social media, papal infallibility, mediatization of religion

## Abstract

This paper is interdisciplinary and combines the research perspective of medical studies with that of media and social communication studies and theological studies. The main goal of this article is to determine [from arguments on all sides of the issue] whether, and to what extent, statements issued by a religious authority can be used as an argument in the COVID-19 vaccination campaign. The authors also want to find answers to the questions of how the pope’s comments affect public opinion when they concern the sphere of secular and everyday life, including issues related to health care. The main method used in this study is desktop research and the analysis of the Roman Catholic Church’s teaching on vaccination and on the types and significance of the pope’s statements on various topics. The auxiliary methods are sentiment analysis and network analysis made in the open source software Gephi. The authors are strongly interested in the communication and media aspect of the analyzed situation. Pope Francis’ voice on the COVID-19 vaccination has certainly been noticed and registered worldwide, but the effectiveness of his message and direct impact on Catholics’ decisions to accept or refuse the COVID-19 vaccination is quite questionable and would require further precise research. Comparing this to the regularities known from political marketing, one would think that the pope’s statement would not convince the firm opponents of vaccination.

## 1. Introduction

This paper is embedded in a research trend related to the influence of religion and the role of religious leaders with regard to public health concerns [1,2,3]. It is also part of current research on the influence of religion on decisions about accepting or rejecting vaccinations (against various diseases; the problem had existed before the outbreak of the COVID-19 pandemic). Nonetheless, very few studies have focused on the Catholic religion, nor have they addressed papal comments on vaccination. The main method used in this study is the analysis of the Roman Catholic Church’s teaching on vaccination and on the types and significance of the pope’s statements on various topics. The auxiliary methods are sentiment analysis and network analysis made in the open source software Gephi. In this case, the specific object of study is the emotions (sentiment) evoked among Twitter users by the remark of Pope Francis about the moral necessity of vaccination against COVID-19.

The data from Twitter cover the period from 6 January 2021 to 21 February 2021 (after Pope Francis expressed his opinion on the vaccine and in the time around this event).

Limitations: One of the obligatory settings was the selection of tweets without a specified language (I)—the number of tweets in Polish was negligible and could not provide a basis for the analysis. The advantage (II) of this approach was the use of the tidytext library, which offers the possibility to use sentiment dictionaries—a prepared database of words with an assessment of their sentiment in English; there was a need for detailed text processing, as the text contained undesirable characters (the details are described in the subchapter Data preparation), any other words in other languages entered along with the search terms could not be measured in terms of the sentiment level (III) The rtweet library and the obtained API-developer configurations were used to collect data from Twitter.

In detecting communities by tracing the discussion, a notable limitation is the scope of the search filter. Tweets under discussion are captured after a word search is made on the Twitter API application, for example, “pope + vaccine”. However, various alternative versions of the string with # were applied, for example, “#pope + vaccine “, “#popevaccine”, etc.

Human coronaviruses were first discovered in the 1960s [4]. They caused epidemics in East Asia and the Middle East. In 2002, cases of severe acute respiratory syndrome (SARS-CoV) were reported, as well as a respiratory syndrome (MERS-CoV) in the Middle East in 2012 [5]. They were identified as more infectious and causing life-threatening diseases among infants, the elderly and those who are immunocompromised [6], as compared to other types of coronaviruses that trigger the symptoms of the ‘common cold’ [7].

The new coronavirus of severe acute respiratory syndrome 2 (SARS-CoV-2) was originally identified on 12 December 2020 in Wuhan city, Hubei province, China, where an outbreak of the epidemic was reported. The current pandemic caused by SARS-CoV-2 is the third consecutive human CoV outbreak in the last two decades [8]. The COVID-19 virus disease poses a global public health threat of the 21st century [9,10], which has far surpassed SARS and MERS in numbers and territorial coverage [11], spreading rapidly worldwide [10,12,13]. SARS-CoV-2 has a lower mortality risk compared to SARS-CoV, but according to epidemiological data, it is more easily and rapidly transmitted, and due to the long incubation period of the virus, as well as the asymptomatic course of the COVID-19 disease, it is unexpectedly difficult to identify and eliminate the virus [14]. The SARS-CoV-2 virus predominantly infects the respiratory tract, causing symptoms ranging from mild to severe acute respiratory syndromes combined with organ failure in some patients, and in certain cases leading to death [15]. The most common symptoms of the infection are fever, cough, shortness of breath, and overall fatigue. In severe cases, systemic infections and pneumonia also coexist [11].

From 4 March 2020 to 22 October 2021, there were 2,961,923 infections and 76,359 deaths [16]. A total of 242,822,630 infections and 4,935,086 deaths were reported worldwide. The highest number of infections (45,352,376) and deaths (734,550) were reported in the USA. In Europe, Russia ranks highest with 8,041,581 infections and 224,369 deaths due to COVID-19. Poland ranks 35th in the world according to the COVID-19 Dashboard by the Center for Systems Science and Engineering (CSSE) at Johns Hopkins University [17]. All the above data are from 22 October 2021.

At the start of the SARS-CoV-2 pandemic in 2020, there were no clinically approved vaccines, so the only protection against infection, according to WHO recommendations, were personal preventive behaviors of social distancing, wearing protective masks and disinfection, along with public health interventions including testing for SARS-CoV-2, monitoring infections and deaths, and implementing countermeasures, such as restrictions on social meetings [11]. At present, vaccines against SARS-CoV-2 are available and in widespread use. There is a decrease in the number of cases as the vaccination rate is increasing. As of 22 October 2021, there were 6,760,365,712 vaccinations registered worldwide, including 38,510,829 in Poland, of which 1,409,652 were vaccinated in the last 28 days [17].

Vaccines differ primarily in their mechanism of action. The process of developing vaccines is long and multi-staged. In the fight against COVID-19, two research phases of the vaccines were accelerated: the preclinical and the clinical ones. WHO data show that as of early January 2021, 63 vaccines were in clinical trials and 172 in preclinical studies [18]. The first vaccine approved in the EU countries (21 December 2020) was produced by Pfizer/BioNTech (Comirnaty) [19]. It consists of single-stranded mRNA, coated with lipid nanoparticles, which encodes the full length S protein of the SARS-CoV-2 virus. The expression of mRNA in host cells is transient. Two nucleoside point mutations are introduced into the mRNA to encode proline, which causes the emerging S protein to adopt a pre-fusion conformation [20]. The product is suitable for patients over 16 years of age. It requires two doses with at least 21 days’ gap between them. The efficacy after both doses is 95%. The storage temperature for this vaccine is from −90 °C to −60 °C [19,21].

The next vaccine approved for use was a product by Moderna (6 January 2021) [22]. This vaccine, similarly, to the Pfizer preparation, is an example of a genetic vaccine. It is administered to adults (over 18 years of age) in two doses at least 28 days apart. It has an efficacy of 94.1% [23]. It can be stored for 7 months at −25 °C to −15 °C [21].

By decision of the European Commission of 21 January 2021, the next vaccine to be launched on the market was a product by Astra Zeneca called Vaxzevria [24]. This vaccine contains a recombinant, non-replicating chimpanzee adenovirus (ChAdOx1) encoding the SARS-CoV-2 virus S protein. This particle serves as a vector that carries the SARS-CoV-2 spike protein, resulting in immune system response [25]. The vaccine is recommended for people aged 18 years and older and is applied in two doses, 28 to 84 days apart. Its efficacy is 59.5% and the storage temperatures are 2–8 °C [26].

The vaccine of the Janssen Pharmaceutica company was authorized with a Conditional Marketing Authorisation in the European Union on 11 March 2021 [27]. It is also based on vectors—active viruses that reduce the risk of infection. The vaccine mobilizes the immune system to produce antibodies. Unlike those described above, it requires a single dose [28]. The Gam-COVID-Vac (Sputnik V) vaccine, developed at the Gamaleya National Research Centre for Epidemiology and Microbiology, was registered in Russia on 25 August 2020. Similar to the preparations from Astra Zeneca and Janssen Pharmaceutica, the Russian vaccine is based on adenoviruses, which are vectors. This vaccine has a 91.6% efficacy [24,28,29]. According to the EU Certificate of Vaccination, it is possible to use mixed schedules with the vaccines Vaxzevria (AstraZeneca), Comirnaty (Pfizer BioNTech), Spikevax (Moderna). The decision is made by the doctor qualifying for vaccination, taking into account the best interests of the patient [30].

The data as of 22 October 2021 suggest that the European Medicines Agency is conducting a phase review procedure for four vaccines: the recombinant protein vaccine (NVX-CoV2373), the vector vaccine Sputnik V (Gam-COVID-Vac), the inactivated vaccine with adjuvant Sinovac, and the protein vaccine with adjuvant Vidprevtyn (Sanofi Pasteur, Lyone, France) [31]. Currently, all people over the age of 18 can receive another dose of the Pfizer or Moderna vaccine 6 months after completing the basic vaccination schedule [32].

COVID-19 vaccines are being developed rapidly compared to traditional vaccines and are approved worldwide through the Emergency Use Authorisation (EUA). The distribution of effective and, above all, safe vaccines is a priority for all countries in the fight against the COVID-19 pandemic [33].

Pope Francis’ position on COVID-19 vaccination has been unequivocal since a number of questions have been raised about the development, testing, and administration of the vaccines.

At the beginning of January 2021, Pope Francis expressed the view that vaccination was a moral obligation because one’s own health and life as well as the lives of others were at stake, and that rejecting vaccination was “suicidal denialism”. The Vatican announced at the time that it would be launching a vaccination campaign among its employees and their families in the middle of the month—a total of more than 10,000 people were estimated to join [34]. Pope Francis himself was vaccinated as soon as the vaccination campaign began [35]. In August 2021, he repeated the appeal to receive vaccines against the SARS-CoV-2 virus, calling vaccination an “act of love” [36]. The seriousness with which the issue of vaccination was taken can be illustrated by the fact that in February 2021 a decree was issued requiring Vatican employees who refused to receive the vaccine to present a medical document explaining the refusal, otherwise facing various consequences, including termination of employment. After criticism and outrage, the Vatican abandoned this rigorous provision, saying that “alternative solutions” would be prepared for those who did not wish to be vaccinated and that “freedom of individual choice” would be respected; nevertheless, the whole situation clearly showed a deep understanding and strong support for the vaccination campaign [37]. It is worth mentioning (although this is an event beyond the timeframe of the study) that on 18 September 2021, a decree signed by Cardinal Giuseppe Bertello, the President of Pontifical Commission for Vatican City State, was published. Under its terms, as of October 1, entry to the Vatican would be permitted only to persons who hold a Vatican COVID passport (“Green Pass”), an EU COVID certificate, or another foreign document certifying vaccination against COVID-19 or having had the SARS-CoV-2 disease. A negative test result for SARS-CoV-2 would also authorize entry to the Vatican. The action was based on the personal wish of Pope Francis, expressed during an audience on 7 September [38,39,40].

For a better understanding of the context of the events analyzed, it would also be useful to refer to the following issues:

1. What position has the Catholic Church taken on vaccinations and vaccines so far?
2. Were Francis’ statements on COVID-19 vaccinations infallible, as defined by the Catholic dogma of papal infallibility in matters of faith and morals? To what extent are these statements binding on Catholics?
3. Is the analysis [using Big Data tools] of the discussion that took place on Twitter in relation to the papal statements cited above a reliable reflection of opinion, and to what extent? How does it relate to the theory of mediatization and how does it express the mediatization of religion?

An extensive introduction of the above can be found in Appendix A. The same item numbering in the Bibliography has been used for both files.

This paper is interdisciplinary and combines the research perspective of medical studies with that of media and social communication studies and theological studies. This is due to the subject and scope of the research, the research questions formulated and the objectives of the paper. The main goal of this article is to determine [from arguments on all sides of the issue] whether, and to what extent, statements issued by a religious authority can be used as an argument in the COVID-19 vaccination campaign. The main aim of the paper was to measure emotions, words used, and phrases related to the event using textual data analysis techniques.

The authors also want to find answers to the questions of how the pope’s comments affect public opinion when they concern the sphere of secular and everyday life, including issues related to health care. This implies further questions: As far as the pope is concerned, are his statements on this issue binding for the faithful, and to what extent? Are such statements covered by the dogma of papal infallibility, as understood in the theological sciences, and to what extent? What emotions are aroused in the public space by such statements? What factors weaken or strengthen this message? The answers to these questions will contribute to a better understanding of the mechanisms of social reactions related to the threat to public health.

No hypothesis has been formulated as the research is of an exploratory nature.

## 2. Materials and Methods

### 2.1. Sentiment Analysis

#### 2.1.1. Source of the Data

The analysis was performed on data from Twitter. The data include 1803 tweets related to the phrase pope vaccine from the period 6 January 2021–21 February 2021. The data were collected systematically using the Twitter API (the name of the application created to download the tweets: textyApp—in accordance with the access obtained from the developer.twitter.com website (accessed on 21 October 2021), including the access key and access secret) and with the R language and the rtweet library. The function that was used to retrieve the tweets was search_tweets() configured with the accesses and parameters obtained.

#### 2.1.2. Data Preparation

The data to be analyzed were prepared properly by:

The first step was the unification to lower case letters (all the sentences used in the tweets were converted to lower case)

Then, using the cSplit() function from the splitstackshape package, the sentences were broken down into individual words.

The next step was to remove punctuation marks, such as full stops, commas, semicolons, brackets, question marks, exclamation marks, quotation marks and prefixes, such as http, https, t.co. This was done using the gsub() function. Next, words that are considered redundant in English were loaded from the stopwords package and filtered out with the anti_join() function from the dplyr package.

The analysis of the tweets consists of several parts. The first one involves checking which words in particular posts are charged; for this part, sentiment analysis was used, namely the tidytext library, which, when properly configured, helped to determine words considered positive and negative in English.

In the next step, it was checked which words occur most frequently together; to obtain this, the tm() and tidytext() libraries were used. From the resulting set of words, the most frequent two-element configurations (bigrams) and three-element configurations (trigrams) were checked. In the next step, the focus was on the determination of emotions connected with the use of sentimentr and dplyr libraries. Next, the strongest word associations were checked in the published posts where the correlation of words was the highest—the widyr library was used for this.

Basic statistical operations, such as calculation of keyword count, words occurring together (n-grams) were presented on the data. In order to perform the analysis and measurement, the R language and libraries (e.g., tidytext, ggplot, dplyr, ggraph) were used. The sentimentr library was used to analyze emotions.

Each post was separated into individual words with their identification numbers and properly prepared and cleaned according to the steps mentioned above.

For sentiment analysis, the tidytext library was used, and the function that was used to retrieve sentiments was get_sentiments() with the bing parameter. As a result, it was possible to retrieve words considered negative and positive and combine them with the collected database of tweets. Thanks to this, every word in the tweet was checked for its sentiment.

## 3. Results

**Figure 1:**
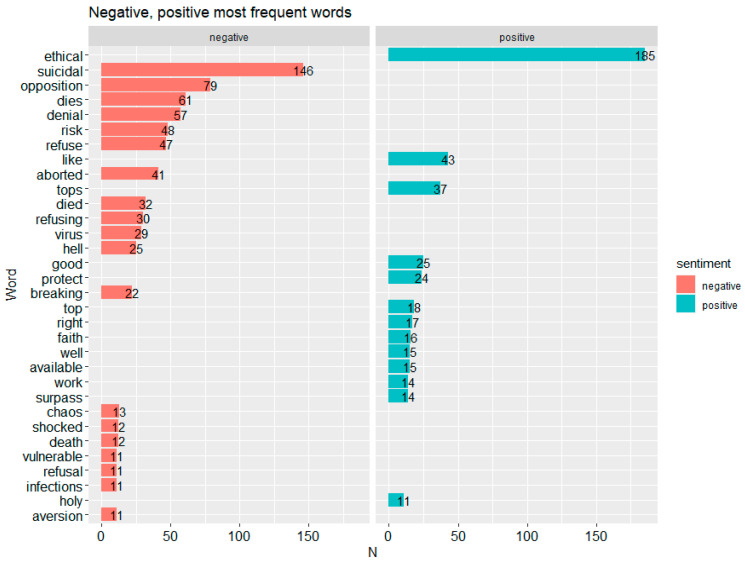
Most frequent words by type of sentiment. Made using the ggplot2 library in R (https://ggplot2.tidyverse.org/authors.html, accessed on 10 December 2021). The red bars represent sentiments found in tweets classified as negative using the tidytext library, while the blue bars represent positive tweets.

Figure 1 shows that the words which were charged most frequently were ethical—185 times (positive), suicidal—146 (negative), opposition—79 (negative). After the words with the highest count, it is clear that negative words have the highest count.

**Figure 2:**
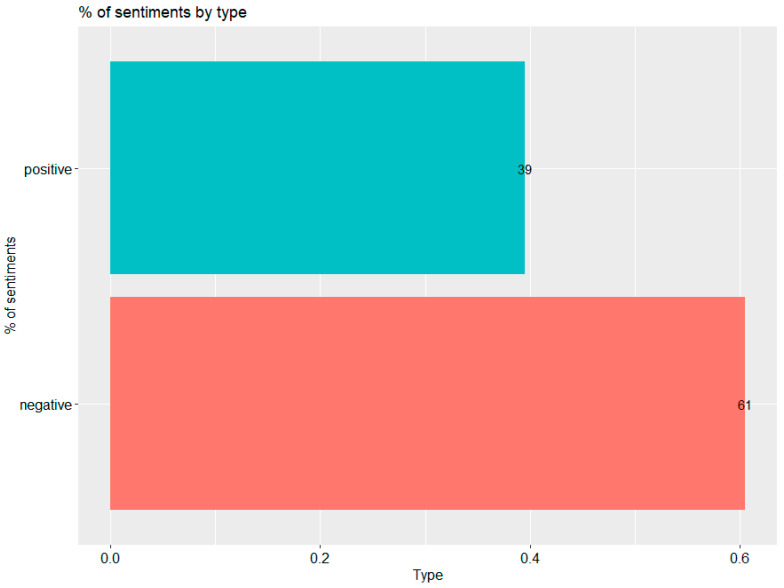
Percentage of sentiments in all the words used in the tweets. Made using the ggplot2 library in R. The red bar shows the percentage of sentiment found in tweets classified as negative using the tidytext library, the blue bar shows the percentage of positive words in tweets—the percentage view.

Figure 2 shows the overall percentage of all words used in prevalence for negative words: 61% compared to 39% of positive words. This shows that the overall attitude towards the event may be rather negative. Further analysis shows other views of the data.

**Figure 3:**
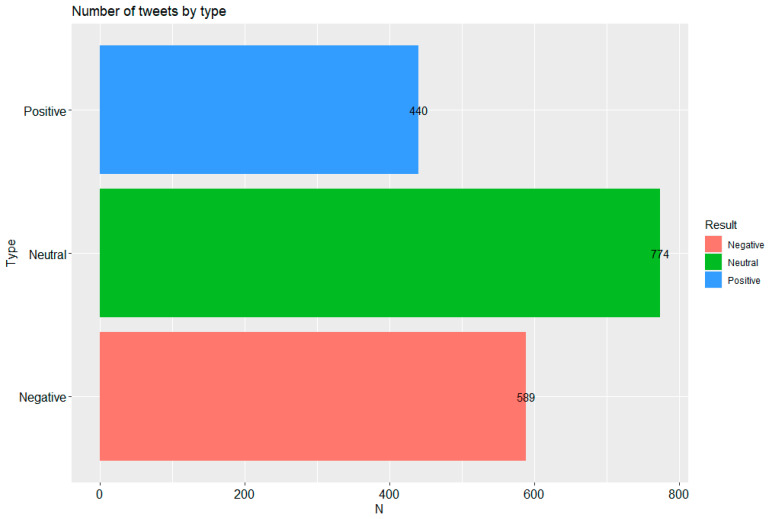
Number of tweets according to type. Made using the ggplot2 library in R. The green bar shows the number of tweets that do not contain charged tweets or the number of tweets for which the number of negative words used in the sentence equals the number of positive words. The blue bar shows the number of tweets in which the positive words used constitute the majority in the tweet, while the red bar is the opposite (the bar shows the number of tweets in a sentence in which the negative words used constitute the majority in that tweet).

In the next step, all the words grouped into individual posts were joined without the unnecessary elements described in the section on data preparation so as to obtain whole sentences without the mentioned elements. Figure 3 shows the tweet count by type according to the adopted algorithm: a tweet was positive if the number of words included in it was mostly positive, while it was considered negative if the number of negative words was predominant. If the words classified as negative and positive balanced each other or were absent, the tweet was classified as neutral. Figure 3 shows that the largest number of tweets were neutral: 774, with 589 negative and 440 positive tweets. Figure 3 also confirms that despite the highest number of neutral posts, more tweets contain negative words.

**Figure 4:**
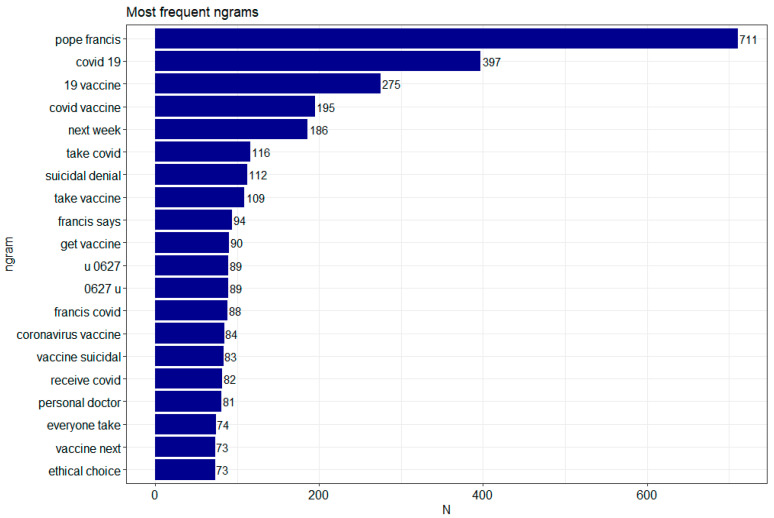
The most common bigrams. Made using ggplot2, tm and tidytext libraries in R.

**Figure 5:**
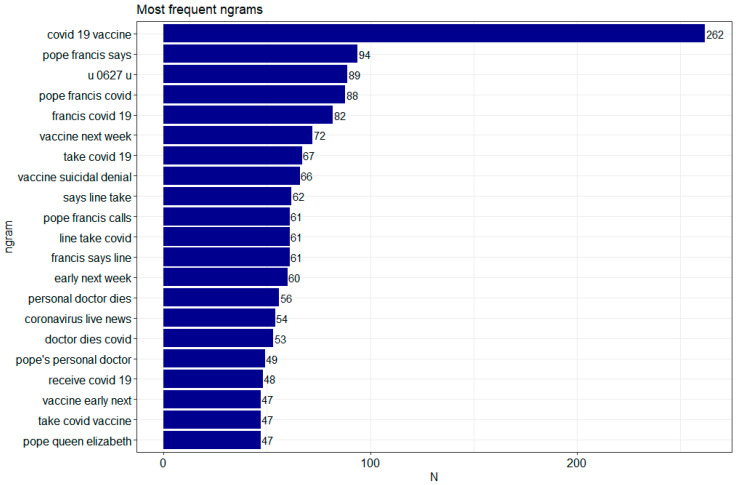
The most common trigrams. Made using ggplot2, tm and tidytext libraries in R.

Since words alone may not be sufficient, it was checked which word configurations occur next to each other in each sentence. The tm() and tidytext() libraries were used to achieve this effect. Configurations of co-occurring words were checked thanks to the n-gram language model. From the obtained corpus of words, the most numerous two-element word configurations (bigrams) and three-element configurations (trigrams) were examined. Figure 4 and Figure 5 show the configurations of words most frequently occurring together. In the case of double words (bigrams), the most frequent configurations are pope francis—711, COVID-19—397, 19 vaccine—275. In the case of triple words (trigrams) the most frequent configurations are: COVID-19 vaccine—262, pope francis says—94, u 0627 u—89 (the configuration is an Arabic letter: Unicode Character ‘ARABIC LETTER ALEF’) [41]. For both bigrams and trigrams, there are those that are either common sense or not charged in any way, such as next week; yet, in prominent positions in the top 10 in both configurations there are, for example, suicidal denial, take vaccine, get vaccine, vaccine suicidal denial, which significantly oppose each side, thus confirming that the event generates considerable emotions.

### 3.1. Emotions

**Figure 6:**
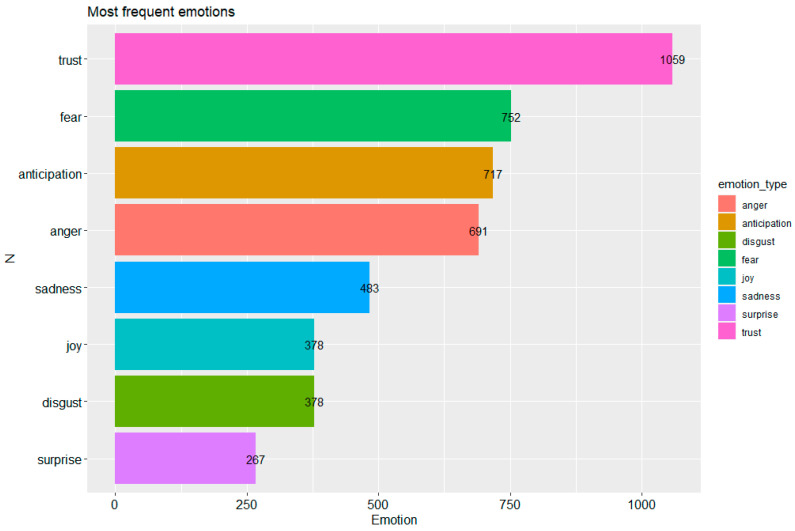
The most frequent emotions. Made using the ggplot2 and sentimentr libraries in R.

This section focuses on identifying the emotions associated with the posts published. The words used in the tweets were combined with the use of the sentimentr and dplyr libraries to obtain statistics about the emotional charge in the tweets. The function used to obtain words that have a sentiment is emotion(). Figure 6 shows that the most frequent charged words are related to emotions: trust—1059, fear—752, anticipation—717. The top ranked emotion is trust; the other seven most frequent emotions are positive: trust (1059) + anticipation (717) + joy (378) + surprise (267) = 2421, while negative emotions, such as fear (752) + anger (691) + sadness (489) + disgust (378) = 2310, so there is a slight predominance of positive emotions (the difference is 111/2421 < 5%).

### 3.2. Correlations

The correlation between words was described as an indicator of how often words appear together as compared to how often they appear separately. The Phi coefficient was used for this [42,43]. The widyr library and the pairwise_cor() function were used to calculate this statistic. This part is to show which words have the strongest association in the total posts published.

**Figure 7:**
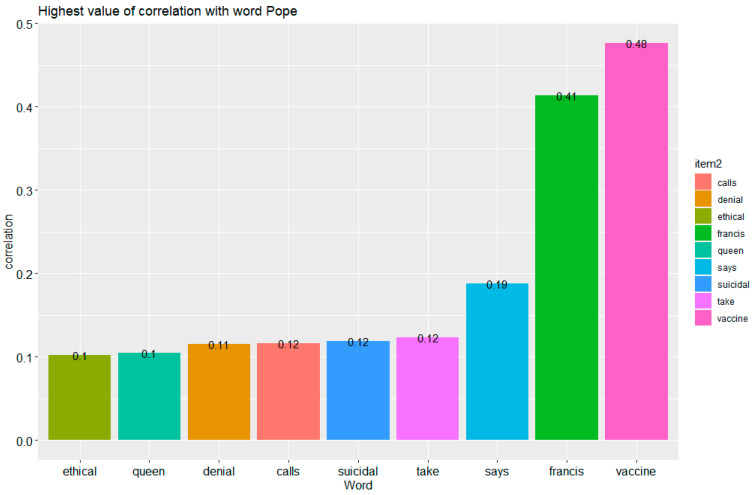
The highest correlations with the word pope. Made using ggplot2 and widyr in R.

Figure 7 shows the highest correlation values with the word pope; in this dataset, these are: vaccine—0.48, francis—0.42, says—0.19.

**Figure 8:**
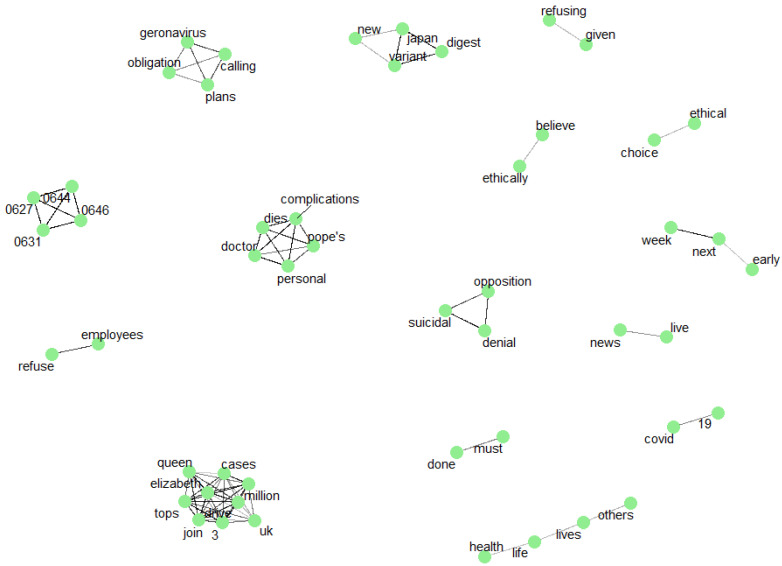
The correlation of words above R > 0.5. Made using ggraph and widyr in R.

Subsequently, other strongest word links were checked where the Phi coefficient correlation was above 0.5. Figure 8 shows the correlation of words above R > 0.5. The words cluster together showing some thematic messages. On the left side of the figure, there are Arabic symbols encoded as numbers.

The correlations of words that provide negative emotions are noteworthy: without medical, other lives life health gambling, suicidal denial opposition (they show significant aversion to vaccines), as well as positive ones: ethical choice, ethically believe.

### 3.3. Detecting Communities

**Figure 9:**
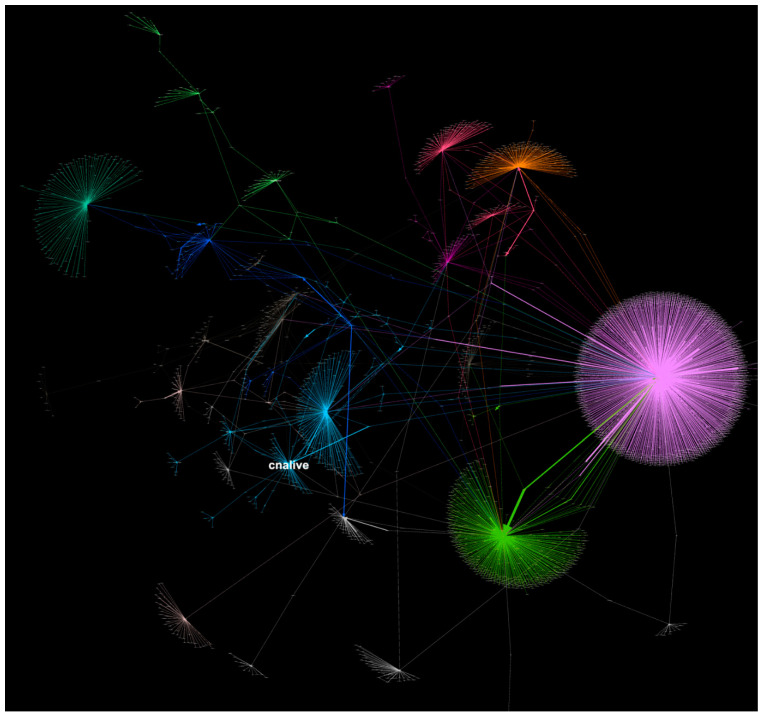
Twitter mentions graph (created in Force Atlas 2 and Fruchtenman Reingold layouts) represents Catholic and lay communities engaged in dissemination and discussion surrounding the Pope’s announcement. The initial announcements from Pontifex (Pope Francis Twitter’s account) and Catholic News Agency—represented by the blue areas in the centre—are re-broadcasted by large international news networks, such as CNN, Reuters, CBS, NBC, ABC, represented by purple, green orange and red areas to the right.

**Figure 10:**
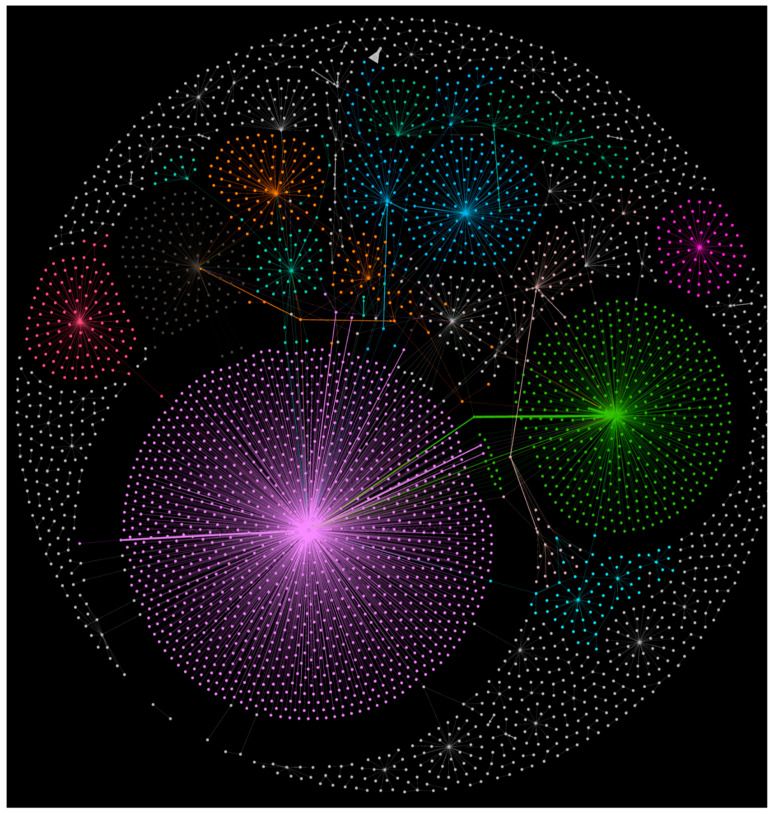
Fruchtenman Reingold Twitter retweets graph reveals the prominent role of CNN (in purple and light green) in redistribution and embracement of the Vatican’s announcement. Catholic news sources are at the top of the graph (blue and teal); communities from Brazil and the Philippines in top left.

Our text sentiment analysis of the gathered corpora of tweets was complimented by network analysis of the data. Such analysis, made in the open-source software Gephi, helps in detecting communities clustered around the prominent Twitter accounts which disseminate Pope’s message on COVID-19 vaccinations. Just over 4000 tweets were gathered to reveal social media affiliations of those engaged in the conversation across the Twittersphere. Community detection algorithms analyze dynamics of the mechanism of mentions when a user mentions another user’s name in his/her own tweet, and retweets, when one tweet is quoted in another tweet. The core feature of the community detection tools is the degree centrality algorithm which detects the position of a node within the network and its distance to other networks (Figure 9). Modularity measures the strength of connections within the network and consequently partitions the emerging network into modules and clusters [44]. We were able to reveal a relatively balanced distribution of the Pope’s announcement across social media and also a balanced and healthy engagement in conversations that followed the Pope’s updated and official stance on vaccinations. This needs to be considered a rare phenomenon in which announcements within the Catholic Church, even on a global scale, are met with equal redistribution in the broader lay community. The Fruchtenman Reingold network graph, best fitted for analysis of medium to large networks, illustrates such a phenomenon on its circular map (Figure 10) where the main sources of redistribution of Pope’s message are clustered into their respective communities of followers and those who mention the news in their own tweets. Catholic News Agency, Vatican News, official Pope Francis’ account of Pontifex, accounts affiliated with Jesuit order (the Jesuit press outlet America Magazine, the account of Jesuit priest James Martin, SJ) appear to be the key players within Catholic communities and news distribution. CNN, CNN International and other major global news outlets are major redistribution sources of the Vatican announcement for the lay parts of the Twittersphere. Some other important nodes of the network emerge: there is a strong presence of Catholic Twitter communities in the Philippines reacting to the coverage from the portal enquirer.net with over 3 million followers on Twitter and from Cnn Philippines (1.2 million followers), and a visible cluster around a single account of a prominent Brazilian journalist Jamil Chade (over 100,000 followers) who embraced Pope’s announcement.

From the moment the major, global news corporations broadcasted the Pope’s message on their live channels and Twitter accounts, Pope’s declaration became part of the global discussion on the urgent topic of vaccinations. Interestingly, our analysis was not able to detect major opponents of vaccinations, discussions tend to be clustered around the respective news sources for communities on Twitter and no major unofficial and self-proclaimed opponents of vaccinations were able to voice their stance loudly. In this sense, perhaps due to the urgency of the global health crisis, the distribution of Pope’s message on the Twittersphere bears traces of the old, top to bottom news distribution paradigm in which announcements coming from official institutions are met with acknowledgment rather than contest and counter-arguments typical to the networked, many-to-many model of news distribution in digital environments [45]. In line with these findings is also the absence of “hashtag hijacking” [46]—a common diversion tactic on Twitter when a prominent account’s message is being retweeted, mentioned and redistributed in order to divert the attention from the original source to the (unrelated) user who accumulates attention (likes, retweets and mentions) at the former’s expense. In other words, community detection algorithms of social network analysis suggest that the Pope’s embracement of COVID-19 vaccines was met with general acclaim and with not much controversy.

## 4. Discussion

When interpreting the results of the study, it is worth considering a few threads forming the background of the pope’s statements, which may have an impact on this interpretation. The first one is the structure of the study group, for example in terms of attitude to vaccination or religion. The second one is the power play of various forces around COVID-19 vaccines, among which the most powerful are the anti-vaccine movements and Russian propaganda. The last issue is the level of public trust in vaccines and the general erosion of authority in the post-truth era.

### 4.1. Twitter Users

When interpreting the results, the structure of the research group is worth attention. Unfortunately, the analyzed data do not contain parameters providing the opportunity to characterize it adequately, therefore, it is only possible to try to make some approximation, based on statistical data from various sources.

The global number of Twitter users in January 2021 reached 353 million [47]. In terms of age structure, the largest group was 35–49-year-olds (28.4%), 25–34-year-olds (26.6%) and 18–24-year-olds (25.2%) [48].

In the global geographical distribution of Twitter users, the top five were the United States (69.3 million), Japan (50.9 million), India (17.5 million), the United Kingdom (16.45 million) and Brazil (16.2 million) [49]. The number of users from these five countries alone amounted to 48% of the global number of users.

For the authors of the study, the religious structure of Twitter users is of interest, as faith affiliation may be reflected in the opinions expressed. Such data are not available, so some approximation was made on the basis of data on the geographical structure of Twitter users and the religious structure of individual countries, thus estimating the number of users representing the Christian religion and the Roman Catholic Church separately. Taking into account the first 20 countries with the largest number of users (together they represent 80.7% of the total number of users), it was estimated that about 33.5% of the global number of Twitter users may be Christians (which, after all, does not deviate from the global share of religions), with about 18.6% being members of the Roman Catholic Church.

These data bring two aspects into consideration when interpreting the results. The predominance of younger age groups is linked to the declining religiousness in many countries today, which may result in less importance being attached to the pope’s statements as an authority. The prevalence of non-Christian religions in the group structure may have a similar effect. These two factors may, therefore, account to some extent for the predominance of neutral or even negative emotions.

### 4.2. Russian Propaganda and Anti-Vaccination Movements

Vaccine hesitancy in online spaces is a phenomenon that had been known and scientifically observed long before the SARS-CoV2 pandemic [50]. Social media are a particularly good arena for spreading disinformation. Social bots (automated accounts impersonating humans) do play a role in magnifying the spread of information by liking, sharing, and searching. The bot population on Twitter has been estimated to range from 9% to 15% [51]. Even before the COVID-19 pandemic, researchers of the vaccine debate on Twitter highlighted the role of Russian trolls, bots and content polluters (spreading malware, unwanted commercial content, etc.). Research suggests that Russian trolls and sophisticated bots tweet much more often than average users about vaccinations and significantly less about vaccine-preventable diseases, promoting both pro-vaccine and anti-vaccine narratives, thus contributing to the strategy of creating political discord [52]. Content polluters also focus primarily on vaccines rather than viruses as a threat, posting anti-vaccine messages 75% more often than the average Twitter user. Such activity could be driven by actual anti-vaccine mood or be a tactic to increase click-through rates by promoting motivational content (clickbait). Similarly, more anti-vaccine tweets are also generated by malicious actors—combinations of bots, trolls and cyborgs [52]. Thus, by creating chaos and deepening social divisions, Russian propaganda aims at destabilizing the situation in Western countries, but it also provides support for the Russian vaccine business. In the context of COVID-19 vaccines, it operates intensively and on a large scale, using narratives ranging from reinforcing conspiracy theories on the origin of the coronavirus, through fueling anti-vaccine sentiment, to promoting the Sputnik V vaccine worldwide [53]. The East Stratcom Task Force, a team established by the EU in 2015 to combat Russian disinformation, added more than 100 examples of pro-Kremlin disinformation content on vaccination to the EUvsDisinfo database in the period January-November 2021 alone [54].

The aforementioned tactic of stoking anti-vaccination sentiment for commercial reasons is supported by the data. The anti-vaxx industry generates annual revenues of at least $36 million and, with 62 million followers on social media platforms, is worth up to $1.1 billion to Big Tech [55]. On Twitter alone, the 2.7 million audience of anti-vaccine activists could translate into up to $7.6 million of annual Twitter revenue by engaging users who are then shown advertisements [55].

The hypothesis that the anti-vaccine movements are responsible for the negative sentiment generated is, however, contradicted by the detecting communities analysis, which did not find activities of a network of trolls or bots in the redistribution of tweets containing the pope’s words. The cited data show what a very difficult research area the topic of COVID-19 vaccination is and how many factors can influence the interpretation of the research results obtained.

### 4.3. Level of Trust in Vaccination

The third theme is the level of trust in vaccination, which can also be reflected in the opinions expressed. Looking at the countries in the top 20 Twitter users, one may notice that levels of trust in vaccination are highly diverse within them. The 2020/2021 survey shows that in the UK, trust in COVID-19 vaccination is at 81%, but there are also countries with high levels of little or no trust—Japan 66%, France 48%, Germany 46%, South Korea 42%, Spain 37% [56]. Data for all countries are unfortunately not available, but even these few examples show that the aspect of trust in vaccination can also be reflected in research results.

The cited data suggest that in the specific topic of COVID-19 vaccination, the opinions expressed should not be linked only to the authority of the pope, because there are many factors that may influence them. The development of social media has undoubtedly contributed to increasing the possibility of studying sentiments in society, but in the case of certain phenomena, in order to understand their causes (and interpret the results of research), it is necessary to consider them in a broader context.

In the analyzed situation of the pope’s statement and the reactions on Twitter, there is probably a tendency in some recipients to refute the pope’s authority if their own feelings and views contradict his; the factors described, such as the intensity of the anti-vaccine movement online or religious differences, as well as the nature of Twitter itself, favor this attitude.

The term post-truth is connected with the exposure of extreme positive and negative values, causing a stronger polarization of views, words and beliefs expressed. In addition, mass media and artificial intelligence algorithms personalize advertisements as well as proposed content and videos, thus fostering the situation when people with certain views are clustered together. Given the fact that the amount of information is increasing at a dizzying pace, it is becoming harder and harder for us as humans to verify the accuracy of the information, which can lead to an easier and simpler way of doing this: taking it for granted as true, especially if it fits our views and confirms our assumptions. This leads to the situation in which we often convince ourselves of the ‘truthfulness’ of our views, irrespective of whether our views are consistent with this truth. This, in turn, may result in a strong defense of one’s views, known as siege mentality [57]. There are camps of people who see the world according to the principle of “we, who know the truth, versus them, who are wrong”. As a result, they ‘bombard’ each other with arguments and use ever-larger verbal ‘cannons’, such as suicidal, denial, without medical.

(IV) If we consider the eight types of most prevalent emotion and classify anticipation as positive, then in this case the differences blur—in favor of positive emotions (V). It is worth investigating whether the phenomenon was constant or whether it changed significantly over time with respect to this event.

COVID-19 Vaccine Hesitancy or Acceptance Rates in different countries reach different levels [58]. Among many aspects, researchers also analyze the reasons for refusal to receive COVID-19 vaccination [59,60]. Possible reasons include distrust of new, unfamiliar preparations and doubts as to their efficacy (no correlation between vaccination rates and infection and morbidity rates) [61]. Social media exposure and interpersonal discussion were also identified as factors influencing the willingness to vaccinate [62]. It was also concluded that the COVID-19 vaccine general beliefs and attitudes were the main determinants of vaccination intention [63]. Moreover, “factors associated with COVID-19 vaccine hesitancy generally mirror factors known to influence vaccine hesitancy for other vaccines. These factors include vaccine-related attributes, political factors, and vaccine-related attitudes and beliefs” [64]. However, there are claims that “attitudes toward the new COVID vaccines may have different sources than attitudes toward vaccines that have been known to the public for a long time” [1]; the factors include a decrease of trust towards doctors and towards science [1]. A general decline in public acceptance of a potential vaccine coupled with increasing vaccine hesitancy might also be the result of pandemic fatigue [65,66].

Religious beliefs are also mentioned among factors in attitudes towards vaccines [2,67]. Previous studies indicate a rather minor role of religious leaders in promoting the acceptance of vaccination [3]. Along with the fact that not all Catholics respect Pope Francis equally (Political Conservatives view Pope Francis as less credible) [68], it should not be surprising to find negative sentiment towards the pope’s encouragement of vaccination.

## 5. Conclusions

The pope, to Catholics, is the authority on matters strictly relating to the faith, but this does not necessarily extend to his teachings in areas involving practical life choices although some individuals in the faith may think his authority does go beyond religious matters. The pope’s encouragement to vaccinate, cannot in any way be regarded as a doctrinal statement concerning faith and morals, either. It should be considered as a consultative voice for those in doubt, seeking support for their decisions in the ecclesial space. Pope Francis’ voice on the COVID-19 vaccination has certainly been noticed and registered worldwide, but the effectiveness of his message and direct impact on Catholics’ decisions to accept or refuse the COVID-19 vaccination is quite questionable and would require further precise research. Comparing this to the regularities known from political marketing, one would think that the pope’s statement would not convince the firm opponents of vaccination. For those who are in favor of vaccination, on the other hand, it will provide an additional argument in the debate. For those who are undecided about vaccination and are looking for arguments to make a decision in the area of their faith, and on top of that acknowledge the authority of Francis, the pope’s attitude may be a factor influencing their decision. The question arises, however, about the real number of such people (it does not have to coincide with the number of people who declare that Francis is an authority for them).

To summarize, some conclusions can be drawn: (I) The event undoubtedly aroused emotion. (II) Words, n-grams and whole tweets all showed that the figures indicated the predominance of negative valence. In many cases, it was due to the use of words with a negative connotation in quotes from the pope himself.

- “It’s an ethical choice, because you are playing with health, life, but you are also playing with the lives of others,” Francis told the station. “I’ve signed up. One must do it.”
- “I don’t understand why some say, ‘No, vaccines are dangerous.’ If it is presented by doctors as a thing that can go well, that has no special dangers, why not take it? There is a suicidal denial that I wouldn’t know how to explain.” [69].
- The pope referred to the vaccination as “an ethical action, because you are gambling with your health, you are gambling with your life, but you are also gambling with the lives of others.” [70].

Other articles:

- Vatican: No punishment for those who refuse COVID-19 vaccine [71];
- Pope’s personal doctor dies from COVID-19 complications, [72]. Excerpt from the text: “Pope Francis’ personal doctor, Fabrizio Soccorsi, has died as a result of ‘complications due to COVID’, the Vatican’s newspaper L’Osservatore Romano announced on Saturday” [72].

(III) The authority of the pope should support a highly positive perception of this event, yet in many cases, statistics show that the opposite is true. This seems to be linked to the term post-truth [57], referring to situations when objective facts are less important in shaping public opinion than resorting to emotions and personal beliefs. The term *post-truth* gained popularity after being named ‘Word of the Year 2016′ by Oxford Dictionaries [73]. However, it has its origins much earlier—in 1992, in Steve Tesich’s comment referring to a person’s free decision to want to live in a post-truth world, and in 2004, when Ralph Keyes developed the idea, pointing to the fact that the notion of lie had become blurred, and that the border between honesty and dishonesty had lost its sharpness. Post-truth is not only a negation of facts but also a kind of permission to go beyond them, by entering the world of emotions, fantasies, often without a clear sense of guilt, with social consent [74]. It is fostered by factors, such as radicalization and populist political rhetoric, the development of new communication technologies where anyone can create and distribute information, succumbing to clickbait culture, being locked in filter bubbles [75].

## Data Availability

Data available on request.

## Appendix A

### Appendix A.1. The Pope and the Vaccination Issue

The question of the authority and position of the pope in the world also fits into the context of this article The pope is both the head of the Catholic Church and the Vatican City State, which is a subject of international law. This means that his actions and comments can be analysed according to different academic disciplines. From the point of view of the Catholic religion, these will be theological studies, but the pope and the Catholic Church are of interest to a wide range of sciences. Sociology studies the Church as a community, a group. It also examines the very phenomenon of religiousness, with the object of research not being the supernatural reality as such, but the ideas that people have about it and their attitudes towards it. From the point of view of management science, one can analyse the structure of the Church as an organisation and study the issue of the Church’s image. The political science of religion has also declared its interest in matters of religion, with its focus on: concepts, structures of religious dogma, religious doctrines and practices that are directly and openly related to politics; religious practices that do not have an explicit political message but can provoke direct political consequences; attitudes of political actors (state authorities, political parties, pressure groups, lobbies, individuals) towards religions and religious communities. Other scientific disciplines that are interested in the Church and religion are for instance cultural studies (the influence of the Church and religion on culture and art, the religious roots of art, etc.), legal sciences, which study the place of the Church in the legal system of individual states, the Church’s in-house canon law, the Church (or rather the Vatican State, whose head is the pope, who is also the earthly head of the Church) as a subject of international law, etc. [76,77].

Undoubtedly, Pope Francis is one of the authorities of the modern world. This is due to the office he holds, but also to his personality traits and his conscious effort to have the Catholic Church perceived as an important player in the international arena, and his voice on important issues (such as migration and migration policy or climate change) widely known and commented on [68,78]. Speaking on important topics, Francis is aware of the ‘media’ power of gestures and sentences [79]. As a result, he is often said to be “the most influential world leader today” [80]. Even if this statement is considered exaggerated, opinion polls show a high position of Francis’ authority both in countries with a high percentage of Catholics, such as Poland [81], and globally [82].

Francis’ statements on the COVID-19 vaccination are embedded in a broader trend in Catholic social teaching concerning the moral judgement of vaccination. This discussion had been going on in the literature long before the emergence of the SARS-CoV-2 virus-it addressed vaccination against other dangerous diseases and the moral judgement of the decision to either receive or refuse vaccines. As Kelly argues, this judgement ought to take into account the fact that “what is rationally optimal for an individual is inherently at odds with the best outcome for the community. It means that if everyone acted out of self-interest with respect to vaccines, communal health would suffer. The Pontifical Council for Justice and Peace’s “four permanent principles” of human dignity, the common good, subsidiarity, and solidarity highlight the issues involved and help ones navigate this significant medical choice with a more informed conscience and a greater sense of their moral responsibilities” [83]. Carson & Flood write directly about “a moral duty to vaccinate” [84]. Neither Catholicism nor most other major religions prohibit vaccinations directly; in fact, there is an indication that “important reasons to vaccinate include preserving health and duty to community” [85]. The fundamental difficulty and moral doubt concerning the acceptance of vaccines (including those for COVID-19) regards cell lines used for the production of vaccines. This problem had appeared in the literature even before the emergence of this disease [86,87,88,89]; as for COVID-19 vaccines, it became the subject of a Congregation for the Doctrine of the Faith statement of 21 December 2021 [90]. Nevertheless, Bednarczyk et al. note that many Catholics hold views contrary to official Church teaching. Thus, it is possible that some Catholics will simply not be swayed by arguments based solely on dogmatic teaching [87].

At this point, the question must be raised as to whether and to what extent these statements of the pope are binding on the followers of the Catholic religion. There is, after all, the dogma of papal infallibility in Catholic theology. It is often misinterpreted, which also results in a misunderstanding of the meaning of various papal statements. It is therefore worth analysing how the pope’s statements on vaccination are interpreted in the light of Catholic theology.

From the perspective of Catholic theology, the papacy is an institution of the Catholic Church, which historically dates back to antiquity and is linked to the place of the martyrdom of the Apostles Peter and Paul-Rome. St Peter’s figure provides the basis for the theological justification for this office, in particular his primacy among the Twelve Apostles and the mission assigned to him by Christ, which was to establish the Church (Matthew 16:16-18) and to strengthen its faith (Luke 22:31-38). Implicatively, each successive Bishop of Rome is the successor of St. Peter in his Roman apostolate and continues his mission, which aims at the continuity and unity of the community [91].

Nowadays this function is called the primacy of Peter and it has the highest form of theological justification-it is a dogmatic definition, promulgated at the First Vatican Council in 1870 and confirmed at the Second Vatican Council. In the ecclesiological aspect, the pope’s authority stems from his supreme jurisdictional power in the Church and from his special charism in transmitting the truths of Christian revelation-infallibility.

This last term is crucial in understanding the importance of the papal voice and its significance in the public sphere. It is important to remember that, in general, papal statements are addressed to members of the Church, but those who prepare them are aware of a much wider audience. This is due to the place of Christianity in European culture and the role of the Church in shaping it [92]. Thus, the authority of papal teaching has a legal and theological aspect, which concerns the faithful, and a pragmatic aspect, which goes beyond the confessional framework. In the latter case, the importance of the institution of the papacy stems from the content value of a given statement, which can influence the shape of public opinion in the secular sphere [93].

In the theological area, the value of papal infallibility is put in organic unity with the infallibility of the whole Church, which has the gift of uncontaminated transmission of the deposit of faith [94]. The starting point for such an approach is the concept of sensus fidei, of which the Second Vatican Council teaches that “the entire body of the faithful, anointed as they are by the Holy One, cannot err in matters of belief. They manifest this special property by means of the whole peoples’ supernatural discernment in matters of faith when ‘from the Bishops down to the last of the lay faithful’ they show universal agreement in matters of faith and morals.” [95].

A particular exemplification of the infallibility of the whole Church is the infallibility of the Pope. The criteria for its existence are as follows:

- the subject of infallibility is the individual pope rather than the Holy See as an institution,
- he speaks ex cathedra, that is, as the supreme shepherd and teacher of all the faithful,
- he consciously engages the full range of its supreme teaching authority,
- he definitively declares a particular truth, thus obliging all the faithful (through a categorical act rather than through recommendations, pieces of advice, admonitions or persuasion); the will for a dogmatic definition must be formally expressed,
- the scope of infallibility is the same as that of the whole episcopate, in the field of faith and morals,
- this teaching, based on the guidance of the Holy Spirit, needs nobody’s approval, nor does it allow any appeal [96].

It is crucial to distinguish between the pope’s ordinary teaching (magisterium ordinarium) and his ceremonial teaching (magisterium extraordinarium, or the solemn magisterium), symbolically called ex cathedra. It is necessary to be aware of the formal aspect of papal statements and their rank. The highest is held by constitutions and bulls, which may contain solemn doctrinal and organisational decrees. Others, as a rule, contain ordinary teaching:

(a) encyclicals—the content of universal character, (b) apostolic exhortations—post-synodal teaching on a particular topic, (c) apostolic letters—occasional writings addressed to particular people, (d) conferences, homilies, speeches and other statements resulting from the everyday pastoral activity. This is confirmed by the fact that the two previous papal ex cathedra rulings (the Immaculate Conception of the Blessed Virgin Mary from 1854 and the Assumption of Mary from 1950) were included in constitutions and bulls.

However, it must be added here that—according to the teaching of Vatican II-divine assistance is also given to the pope when he does not formulate infallible teaching or make definitive declarations, but exercises his ordinary teaching office and gives instructions which lead to a better understanding of Revelation. The faithful ought to adhere to this teaching with the submission of faith [95].

An essential element of ex cathedra teaching is the intention to finally resolve a given issue with an obligation on the faithful to respect that decision. It should be added that this intention should be expressed explicitly and in an unambiguous way. Solemn teaching does not occur, therefore, when the pope speaks as a theologian or as a bishop of the Diocese of Rome [97]. The attribute of infallibility does not pertain to the pope habitually, but currently, that is, in clearly describable situations [98]. In this context, it is worth recalling R. Bellarmine’s statement concerning the theoretical possibility of the pope’s error when he acts as a private person: “A pope who is a blatant heretic automatically (per se) ceases to be pope and head of the Church, since he ceases to be a Christian (Catholic) and a member of the Church.” (as cited in [99]). Therefore, the Church is aware that a pope could become a schismatic if he were to state, in a binding and definitive manner, a doctrine that was contrary to the faith of the Church. And although the law does not know of any authority that could declare this conclusively, if a pope were to fall outside the community of faith because of heresy, it would create a situation parallel to the pope’s death, and the Church would not then be deprived of the possibility of taking action [100].

To conclude this theme, it must be said that both the pope and the whole Magisterium of the Church are conditioned and in a sense bound by the word of God, by the sense of faith of the whole Church, by the Church’s tradition, particularly expressed in the teaching of the Councils, and by the duty to proclaim revealed truth in a way that is accessible to people of a given era and culture [101].

What may be the subject of an infallible papal pronouncement is only the explanation of Catholic doctrine or its defence in matters of faith and morals (doctrina de fide vel moribus). The term morals refers to the natural moral order and the related measures needed to achieve salvation [98]. In other words, these are moral principles that directly stem from faith and constitute doctrina revelata practica. It is, therefore, part of the deposit of faith, containing a moral doctrine belonging to revelation and encompassing content of a practical nature, which is the consequence and implementation of faith in life [99].

With reference to the main problem of this paper, it should be stated that the concern for health, which arises from the Fifth Commandment of the Decalogue, cannot be excluded from this area. The Catechism of the Church says explicitly that “life and physical health are precious gifts entrusted to us by God. We must take reasonable care of them, taking into account the needs of others and the common good.” [102], No. 2288.

The Church has not addressed the issue of concern for health in terms of infallible teaching so far. In this context, it is important to remember John Paul II’s 1995 encyclical Evangelium Vitae. According to some theologians, the pope used a dogmatic formula concerning the protection of life in three places in this document, but a thorough theological analysis excludes this possibility [103,104].

Undoubtedly, the Congregation for the Doctrine of the Faith’s Note on the morality of using some anti-COVID-19 vaccines of 21 December 2020 [105], while having the approval of Pope Francis, is not a document containing infallible Church teaching. Documents of any congregation of the Holy See inherently exclude such teaching. It was issued amid doubts over the moral acceptability of certain vaccine technologies. The document is the provisional voice of the Church and, like all such statements, contains immutable moral principles but is also based on current knowledge, which is subject to the laws of development and change: “We do not intend to judge the safety and efficacy of these vaccines, although ethically relevant and necessary, as this evaluation is the responsibility of biomedical researchers and drug agencies. Here, our objective is only to consider the moral aspects of the use of the vaccines against COVID-19 that have been developed from cell lines derived from tissues obtained from two fetuses that were not spontaneously aborted.” [105].

The pope’s encouragement to vaccinate, contained in a television interview aired on 10 January 2021, cannot in any way be regarded as a doctrinal statement concerning faith and morals, either. It is merely a pastoral encouragement, expressed out of concern for the common good and based on confidence in the widespread medical opinion about the positive effects of vaccination. It can be treated as a testimony encouraging others to action. Neither the formal rank of this statement nor the issues it addresses, with no theological arguments, meet in the least the criteria of teaching ex cathedra. From the theological point of view, therefore, in the context of the whole pontificate of Pope Francis and the forms of his statements, it should be considered as a consultative voice for those in doubt, seeking support for their decisions in the ecclesial space.

### Appendix A.2. Twitter and Social Media as a Mirror of Opinions—Big Data Research as a Way to Understand Social Trends

Social media are now a permanent part of the modern media ecosystem, which is based on multiplatform publishing. They, unlike print, radio and television publishers, cannot be referred to as media broadcasters. Messages are created by producers of goods not associated with the media industry. They do not become mass-media but they benefit from the democratisation of communication, creating a new channel of communication which is independent of the media, or competing with them for the attention of the audience [106]. The social media have become an undoubted rival of the traditional media in fulfilling the informational function. This competition is definitely won by social media among the generation of digital natives [107]. The sender and receiver can constantly switch places; the sender is at the same time the receiver and the other way round, and this happens on a global scale. This specific communication situation means that social media both shape and express public opinion. Therefore, they are a good source of information for researchers of processes, emotions and changes of opinion in society.

Twitter is a microblogging social service founded in 2006. It has gathered nearly 300 million users in less than a decade and now, it is actively used worldwide by 330 million people per month and 145 million per day [108]. With the growth of Twitter and other microblogging platforms, there have been ample opportunities to analyse the data contained there, using Big Data technology. The research is based on sentiment analysis [109,110,111,112,113], which is understood as “the application of natural language, text analysis and computational linguistics to automate the classification of the emotional state of subjective text. It utilizes a variety of big data technologies and concepts including machine learning, natural language processing and cluster computing frameworks tuned for big data processing” [114]. This technique is constantly being improved and finds a wide range of applications.

This includes, for example, research by Johan Bolen, Huina Mao and Xiaojun Zeng, who in 2008 carried out an independent study of the correlation between the value of the Dow Jones Industrial Average stock market index and the content of Twitter posts, analysed in terms of commentators’ moods using two tools: OpinionFinder and Google Profile of Mood States (GPOMS) [115,116]. These authors also proved the opposite correlation: that various events in the public space have an immediate and high impact on public sentiment, which is expressed, for example, by social media posts [117]. Similar research is conducted by Prof. Adam Sadilek from the University of Rochester, who uses the analysis of Twitter statuses to predict the outbreaks of flu [118,119]. Research on predicting the outcome of the US presidential election based on a study of Twitter user statuses was conducted in 2010 by Tumasjan, Sprenger, Sandner and Welpe [120]. Furthermore, Twitter is increasingly investigated as “a means of detecting mental health status, including depression and suicidality, in the population” [121]; it is also explored in a number of other, seemingly different areas such as tourism, dialects on social media, hotel and customer reviews, politics and campaigns, mental health, stock returns and investment, climate change, real estate, movie and product reviews [122].
